# Tumor-Induced Osteomalacia: A Case Report

**DOI:** 10.7759/cureus.63118

**Published:** 2024-06-25

**Authors:** Dayanidhi Meher, Ranjana Giri, Vishal Agarwal, Binod Prusty, Bijay Das

**Affiliations:** 1 Endocrinology, Diabetes and Metabolism, Kalinga Institute of Medical Sciences, Bhubaneswar, IND; 2 Pathology, Kalinga Institute of Medical Sciences, Bhubaneswar, IND

**Keywords:** tumor-induced osteomalacia, symptomatic hypophosphatemia, oncogenic osteomalacia, phosphaturia, fibroblast growth factor-23

## Abstract

Tumor-induced osteomalacia (TIO) is a rare paraneoplastic syndrome with a variable presentation. We present a case of a 55-year-old female who presented with pain in the bilateral hip region for the last two years. On routine biochemical evaluation, she was found to have hypophosphatemia with an X-ray of the bilateral hip region showing an acute stress fracture in the bilateral intertrochanteric region of the femur. An evaluation for the cause of hypophosphatemia revealed renal phosphate loss with low percentage tubular reabsorption of phosphate (% TRP) of 83% (reference range: 85-95%), with tubular maximum phosphate reabsorption per unit glomerular filtration rate (TmP/GFR) of 2.07 mg/dL (reference range: 2.5-4.5 mg/dL (0.67 mmol/L; range: 0.84-1.23 mmol/L)). Further evaluation revealed elevated levels of intact fibroblast growth factor, 445.7 pg/mL (reference range: 23-95 pg/mL). A 68-Gallium DOTA-1-Nal3-octreotide (DOTANOC) PET-CT revealed a focal increased tracer uptake with a lytic lesion at the lateral metaphyseal aspect of the proximal right tibia, suspicious of somatostatin receptor avid mesenchymal tumor, leading to the diagnosis of TIO. Definitive treatment with complete surgical excision of the tumor was done. Postoperatively, her phosphorus level was within the normal target range even without oral phosphate supplementation. While it is a rare condition, a proper and systemic workup can lead to timely diagnosis and management of this debilitating benign condition.

## Introduction

Phosphaturic mesenchymal tumors leading to tumor-induced osteomalacia (TIO) are rare entities in clinical practice [1]. Renal phosphate loss because of elevated fibroblast growth factor-23 (FGF-23) levels is the biochemical hallmark of the disease, which leads to a clinical spectrum comprising nonspecific features such as vague musculoskeletal symptoms such as pain and aches at the pelvic girdle, gait instability, and multiple fractures [2]. These tumors are typically benign and can be found anywhere in the body. It is a rare disease entity with approximately only 1,000 cases reported until today [1]. This might represent a gross underestimation of the actual prevalence of the disease as the signs and symptoms of the disease are usually nonspecific, which may lead to a misdiagnosis by the treating physician [3,4]. Thus, here we describe a case of TIO, who presented with similar nonspecific complaints, and the systemic way we approached the diagnosis of such a case.

## Case presentation

**Figure 1:**
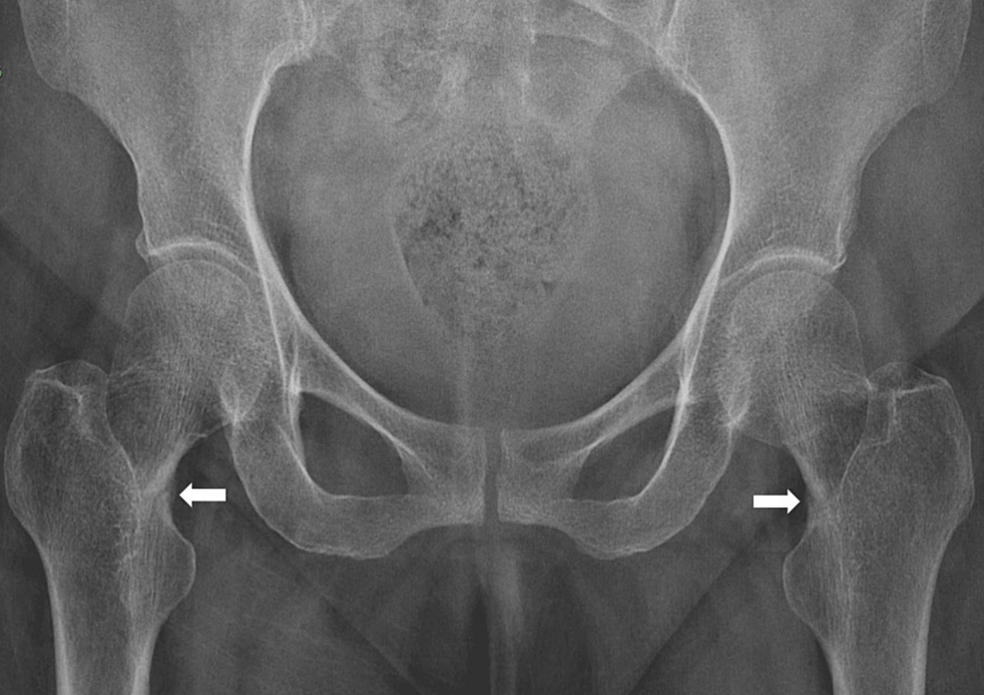
An X-ray of the bilateral hip region showing acute stress fracture (arrows) in the bilateral intertrochanteric region of the femur.

A 55-year-old Asian woman presented to our orthopedic unit with chief complaints of pain in the bilateral hip region for the last two years, along with the inability to walk for the last six months. The patient was initially able to walk without support, but, gradually, she was confined to a wheelchair for the past three months. She had a known case of primary hypothyroidism and hypertension for the past three years and was on telmisartan 40 mg once daily and levothyroxine supplementation 25 mcg once daily. There was no history of trauma and weight loss, and the patient attained menopause at the age of 50 years. A physical examination revealed tenderness on the internal rotation of bilateral hip joints. There was no evidence of any bony or soft tissue swelling. The systemic examination was unremarkable. On routine biochemical evaluation, she was found to have hypophosphatemia with a serum phosphorus level of 1.4 mg/dL (reference range: 2.5-4.5 mg/dL (0.45 mmol/L; range: 0.8-1.45 mmol/L)), with X-ray of the bilateral hip region showing acute stress fracture in the bilateral intertrochanteric region of the femur (Figure 1). A dual-energy X-ray absorptiometry (DEXA) scan of the anteroposterior spine revealed a T-score of -3.5. The patient was referred to the endocrinology unit given the evaluation of hypophosphatemia and osteoporosis.

Evaluation for the cause of hypophosphatemia revealed a renal phosphate loss with a low percentage tubular reabsorption of phosphate (%TRP) of 83% (reference range: 85-95%), with tubular maximum phosphate reabsorption per unit glomerular filtration rate (TmP/GFR) of 2.07 mg/dL (reference range: 2.5-4.5 mg/dL (0.67 mmol/L; range: 0.84-1.23 mmol/L)). A further evaluation for the cause of renal phosphate wasting revealed elevated levels of intact fibroblast growth factor-23 (445.7 pg/mL (reference range: 23-95 pg/mL)). The serum 1,25-dihydroxy vitamin D (calcitriol) level was 43.2 picomoles per liter (pmol/L) (reference range: 47.8-190.3 pmol/L (18 pg/mL; range: 19.9-79.3 pg/mL)). Her biochemical investigations are summarized in Table 1.

**Table 1:** Biochemical investigations at admission day one, day two, day 10, and postoperative day seven. Abnormal values are shown in bold font. Values in parentheses are an international system of units (SI). Abbreviations: WBC, white blood cell; T3, triiodothyronine; T4, thyroxine; TSH, thyrotropin (thyroid-stimulating hormone); FePO4, fractional excretion of phosphorus; %TRP, percentage tubular reabsorption of phosphorus; Tmp/GFR, tubular maximum phosphate reabsorption per unit glomerular filtration rate; FGF-23, fibroblast growth factor-23

Parameter	Day 1	Day 2	Day 10	Postoperative day 7	Normal value
Hemoglobin	12.1 gm/dL	-	-	-	12-15gm/dL
WBC	6,700/µL	-	-	-	4,000-10,000/µL
Free T3	3.52 pg/mL (5.4 pmol/L)	-	-	-	2.8-4 pg/mL (4.3-6.1 pmol/L)
Free T4	1.17 ng/dL (15 pmol/L)	-	-	-	0.8-1.8 ng/dL (10.3-23.2 pmol/L)
TSH	4.168 µIU/mL	-	-	-	0.35-4.2 µIU/mL
Serum albumin	4.4 gm/dL	-	-	4 gm/dL	3.9-4.9 gm/dL
Serum phosphorus	1.3 mg/dL (0.41 mmol/L)	1.4 mg/dL (0.45 mmol/L)	2.5 mg/dL (0.80 mmol/L)	4.1 mg/dL (1.32 mmol/L)	2.5-4.5 mg/dL (0.8-1.45 mmol/L)
Serum calcium	9.2 mg/dL (2.29 mmol/L)	-	-	8.9 mg/dL (2.22 mmol/L)	8.6-10.3 mg/dL (2.15-2.57 mmol/L)
Serum magnesium	2.1 mg/dL (0.86 mmol/K)	-	-	-	1.6-2.6 mg/dL (0.65-1.06 mmol/L)
25-hydroxy vitamin D	39 ng/mL (97.3 nmol/L)	-	-	-	20-65 ng/mL (50-162 nmol/L)
1,25-dihydroxy vitamin D	-	-	18 pg/mL (43.2 pmol/L)	-	19.9-79.3 pg/mL (47.8-190.3 pmol/L)
Parathyroid hormone(intact)	43.4 pg/mL	-	-	-	15-65 pg/mL
Serum creatinine	-	-	0.78 mg/dL (68.9 µmol/L)	-	0.5-1.1 mg/dL (44-97 µmol/L)
Urine creatinine	-	-	68.92 mg/dL 6,092.6 µmol/L	-	20-275 mg/dL (1,768-24,310 µmol/L)
Urine phosphorus	-	-	38.9 mg/gm	-	105-1,081 mg/gm
FePO4	-	-	17%	-	<15%
%TRP	-	-	83%	-	85-95%
TmP/GFR	-	-	2.07 mg/dL (0.67 mmol/L)	-	2.6-3.8 mg/dL (0.84-1.23 mmol/L)
FGF-23	-	-	445.7 pg/mL	-	23-95 pg/mL

**Figure 2:**
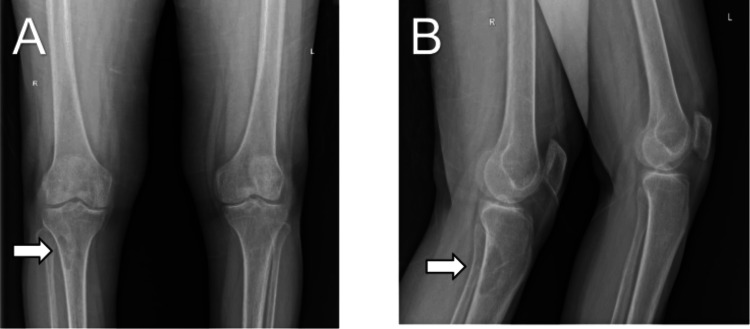
An X-ray of bilateral knee joints showing osteolytic lesion (arrow) in the posterolateral aspect of the right tibia: panel A (anteroposterior view) and panel B (lateral view).

**Figure 3:**
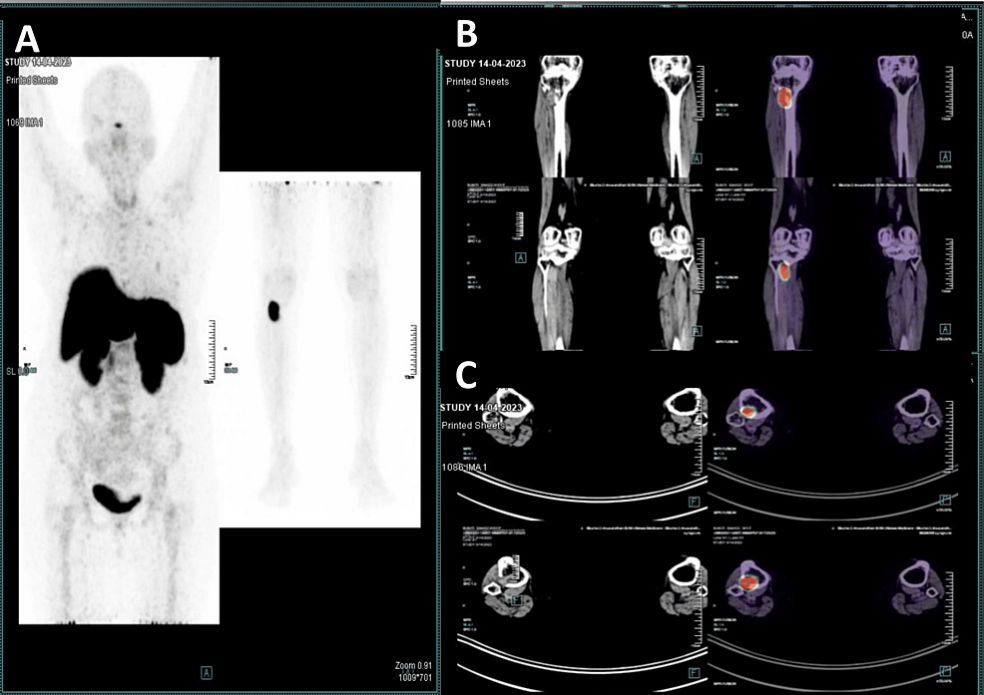
A 68-Gallium DOTANOC PET-CT scan (panel A) showing a focal increased tracer uptake in the metaphyseal aspect of the proximal right tibia (approximate size: 2.1 cm x 2.4 cm x 2.9 cm, standardized uptake values (SUV) max: 23.1). No pathological focal tracer uptake or lytic/sclerotic lesion was noted in the rest of the visualized skeleton. Panels B and C are transverse and coronal views, respectively.

An X-ray of the right knee revealed an osteolytic lesion on the posterolateral aspect of the metaphyseal region of the right tibia (Figure 2). Gallium-68 DOTA-1-Nal3-octreotide (DOTANOC) PET-CT revealed a focal increased tracer uptake with lytic lesion along with a soft tissue component of lateral metaphyseal aspect of proximal right tibia (2.1 cm x 2.4 cm x 2.9 cm, standard uptake value maximum of 23.1), suspicious of somatostatin receptor avid mesenchymal tumor (Figure 3).

**Figure 4:**
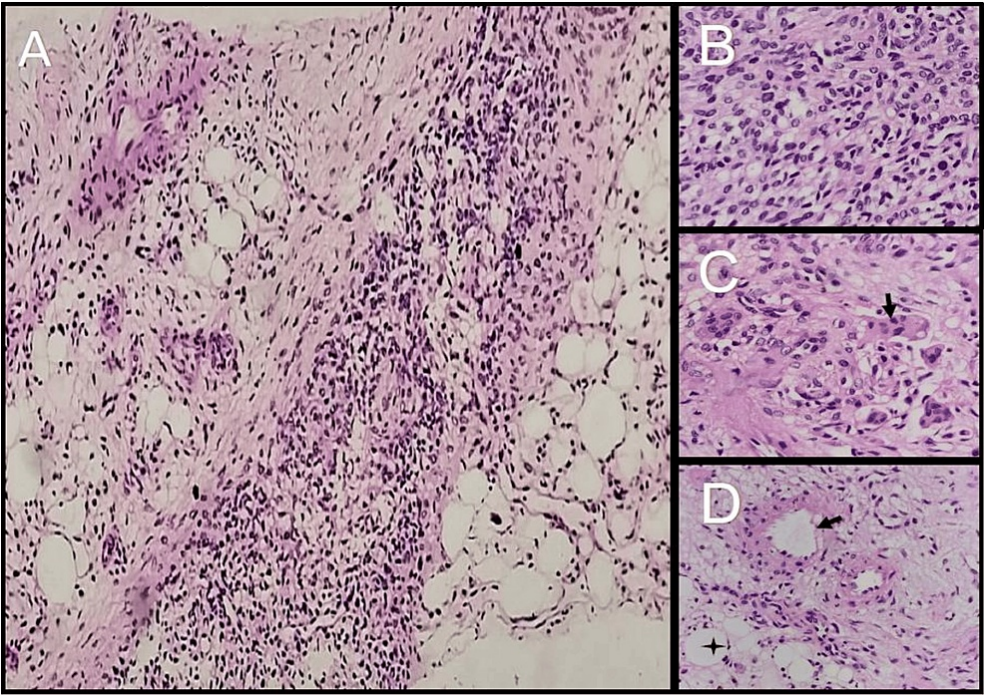
Histological images of the CT-guided linear core biopsy. Panel A showing tumor cells arranged in sheets admixed with adipocytes and blood vessels (100x). Panel B showing bland-looking spindle cells with inconspicuous nucleoli with no nuclear atypia, mitosis, or necrosis (400x). Panel C showing multinucleated giant cells (arrow) (400x) and panel D showing blood vessels (arrow) and adipose tissue (star) (400x). Panels A through D, hematoxylin-eosin staining.

**Figure 5:**
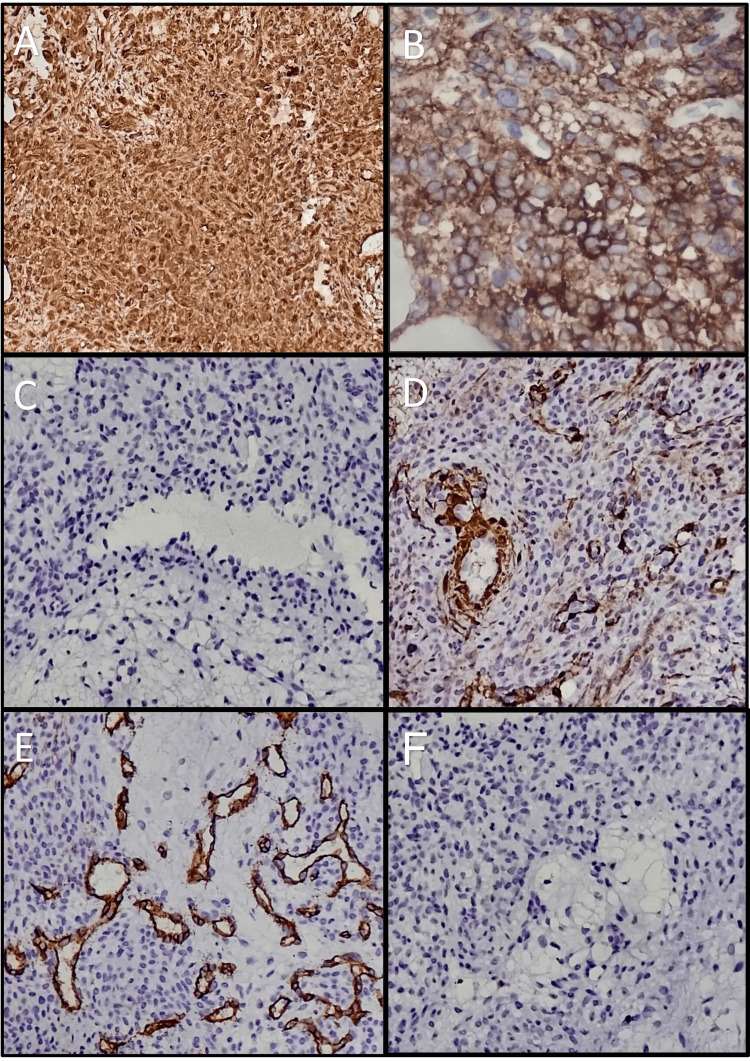
Immunostaining. Panel A and Panel B stain immune-positive for vimentin and CD56, respectively. Panels C, D, E, and F show tumor is immune-negative for S-100, smooth muscle antibody (SMA), CD34, and desmin, respectively.

A CT-guided biopsy of the lesion was done, which showed a phospaturic mesenchymal tumor (Figure 4). On immunohistochemistry (Figure 5), it was positive for vimentin and negative for S-100, smooth muscle antibody (SMA), and CD34. A diagnosis of TIO was made, and the patient was planned for operative management. Preoperatively, the patient was managed with oral phosphate supplementation of 40 mg/kg/day in four divided doses, along with oral calcitriol supplementation at a dose of 0.5 μg/day.

**Figure 6:**
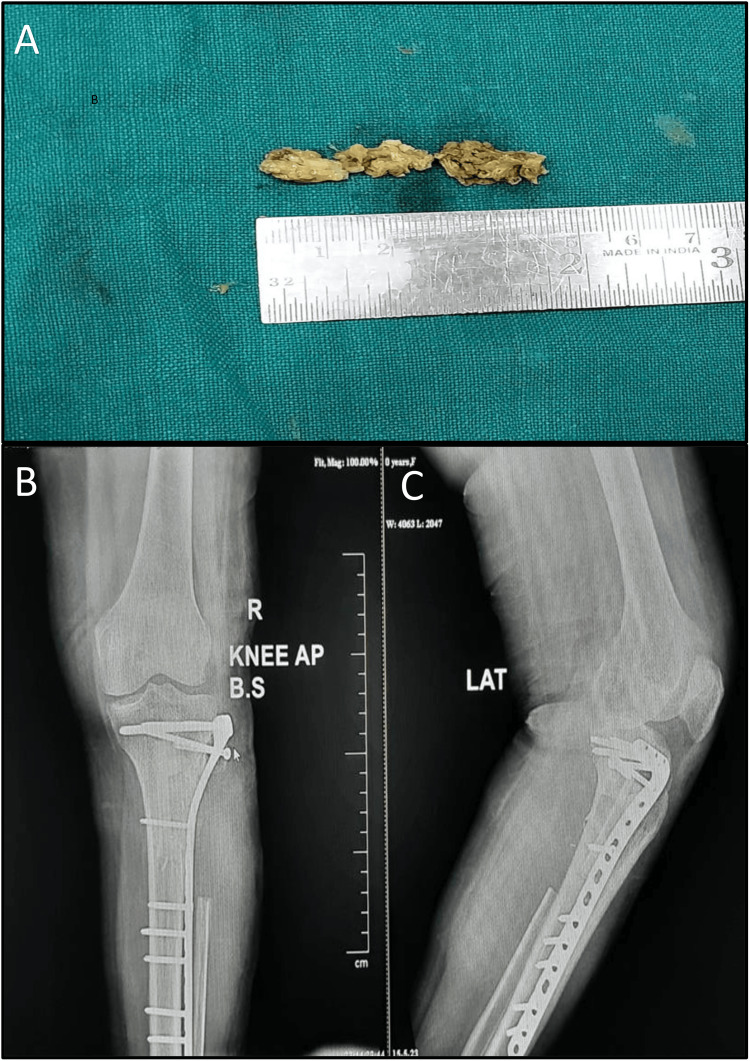
Panel A showing gross tumor specimen, post-excision. Panels B and C showing postoperative X-ray with excision of tumor mass with partial fibulectomy, with stabilization with lateral tibial locking compression plate and tricortical iliac crest bone grafting.

Definitive treatment with excision of the tumor (Figure 6) with partial fibulectomy with stabilization with lateral tibial locking compression plate and tricortical iliac crest bone grafting under spinal and epidural anesthesia was done.

Postoperatively, her phosphorus level was within the normal target range, even without oral phosphate supplementation. At three months and six months after surgery, the patient was reviewed, and the patient was able to walk without support.

## Discussion

TIO is a rare clinical diagnosis often associated with diagnostic delay owing to its nonspecific symptoms. As per the literature, the overall misdiagnosis rate has been reported up to 95%. This report describes a case of TIO in a 55-year-old woman, caused by a phosphaturic mesenchymal tumor in a 55-year-old woman. As per the literature, the usual age of presentation is between 40 and 45 years [3]. In adults, the most common symptom at presentation is usually bone pain, arising at the site of the localized tumor, or because of pathological fractures (80% of patients). Because of delays in diagnosis, the patient’s bone health usually worsens, and the patient usually gets confined to a wheelchair [5]. Symptoms are usually present for several months to years, and the average delay in diagnosis from the onset of symptoms is usually 2.9 ± 2.3 years. As seen in our case, the patient was symptomatic for the past two years and was undiagnosed because of nonspecific symptoms of low back ache and gait instability because of proximal myopathy and stress fractures at the bilateral femoral neck and intertrochanteric region.

The typical biochemical pattern of TIO results from increased fibroblast growth factor 23 (FGF-23), which leads to an increase in renal phosphate leak, subsequently leading to low serum phosphate levels [6]. Serum calcium, 25-hydroxyvitamin D, and parathyroid hormone levels are typically normal, with low or inappropriately normal 1,25-dihydroxy vitamin D levels, owing to the inhibition of 1-alpha hydroxylase vitamin D3 enzyme by FGF-23.

Once the diagnosis is suspected, the presence of hypophosphatemia needs to be established as the first initial step of evaluation [7]. Morning fasting blood specimen for serum phosphate is always preferred as food intake increases serum phosphate levels. Age-specific cutoffs should be taken in the case of children. Once the presence of hypophosphatemia is biochemically documented, the next step in evaluation is to look for the cause of hypophosphatemia. History of intake of antacids may point towards decreased absorption of phosphate. Phosphate loss via kidneys comprises the major cause of hypophosphatemia and is confirmed by the presence of low %TRP and low TmP/GFR [7]. In our case, there was persistent hypophosphatemia along with normal values of serum calcium, serum magnesium, serum 25-hydroxy vitamin D, and parathyroid hormone levels, along with evidence of renal phosphate loss demonstrated by low %TRP of 83% and low TmP/GFR of 2.07 mg/dL. This leads us to look for the cause of renal phosphate leak by measuring serum intact fibroblast growth factor 23 (iFGF23), a phosphaturic hormone that causes renal phosphate loss and has been implicated in having a pathogenic role in TIO [8]. Elevated levels of iFGF23 lead us to the next step of tumor localization. Tumors causing TIO can be present anywhere in the body from head to toe and are usually small and slow-growing [9].

As the physical examination was unremarkable in our case with no history of any bony or soft tissue swelling, we went for functional imaging with whole-body gallium-68 DOTANOC PET-CT to localize the tumor. As per the literature, the lower extremities (59.6%) are the most common site for tumor localization in patients with TIO. In 24% of cases, it is localized in the head and neck region, while the upper extremities and torso comprise 9.4% and 6.9% of cases, respectively [10]. In our case, it was localized to the lower extremity, involving the upper end of the right tibia. The patient later underwent anatomical imaging with contrast-enhanced magnetic resonance imaging (CEMRI) to delineate the local extension of the tumor and to plan for surgical resection.

The definitive management of TIO comprises the complete surgical resection of the tumor mass [1]. Our patient was subjected to surgical resection of the tumor and the postoperative recovery was remarkable as previously discussed.

The differential diagnosis of TIO includes other causes of hypophosphatemia, such as malabsorption syndrome, and nutritional phosphate deficiency, the possibility of which is suspected from the patient’s past medical history and dietary history. The young age of presentation, along with growth retardation and ear or dental abnormalities in the patient or their family members may hint toward genetic causes of osteomalacia associated with elevated FGF-23 and hypophosphatemia. This includes autosomal dominant, autosomal recessive, and X-linked forms of hypophosphatemic rickets. Disorders such as Fanconi syndrome and hereditary hypophosphatemic rickets with hypercalciuria constitute the differential diagnosis when renal phosphate leak is accompanied by low FGF-23 [11].

## Conclusions

TIO can have varied presentations. Owing to its multifaceted presentation, it warrants a multidisciplinary approach to identify and localize the tumor. A complete resection of the tumor mass leads to a cure in such cases. This case highlights the need for proper and systematic evaluation of a case of hypophosphatemia, leading to the diagnosis of TIO.
